# Should prophylaxis against *Pneumocystis jirovecii* pneumonia be considered in selected patients with leprosy reactions?

**DOI:** 10.1186/s41479-024-00127-x

**Published:** 2024-04-05

**Authors:** Nuria Ramírez-Perea, Claudia Moran-Castaño, Jose A. Perez-Molina

**Affiliations:** 1 Internal Medicine Department, Elda University Hospital, Alicante, 03600 Spain; 2 Internal Medicine Department, Carmen y Severo Ochoa Hospital, Asturias, Spain; 3 National Referral Unit for Tropical Diseases, Infectious Diseases Department, Ramon y Cajal University Hospital, IRYCIS, Madrid, 28034 Spain; 4 CIBER de Enfermedades Infecciosas, Instituto de Salud Carlos III, Madrid, 28029 Spain

**Keywords:** Leprosy, Immunosuppression, PJP prophylaxis, Leprosy reactions, Dapsone

## Abstract

Leprosy reactions often require prolonged high-dose steroids or immunosuppressive drugs, putting patients at risk of *Pneumocystis jirovecii* pneumonia (PJP). However, no PJP cases are reported, possibly due to dapsone treatment for leprosy. In patients with leprosy reactions not receiving dapsone because of toxicity or resistance and requiring long-term immunosuppression, PJP prophylaxis should be considered.

## Background

*P. jirovecii* pneumonia (PJP) in the AIDS era, and prior to the use of prophylaxis, affected 70-80% of these patients [1]. In hematological transplant recipients, although clearly lower, incidence was as high as 5-16% [2]. Furthermore, mortality in people with and without HIV was 10-30% and 20-50% respectively [2, 3]. Since the introduction of routine PJP prophylaxis, incidence has declined to <1 case/100 persons living with HIV (PLWH)/year [3] and <1% of hematologic transplant patients [2]. Most information and clinical guidelines for PJP’s prevention have focused mainly on PLWH, oncology patients and transplant recipients. However, there are still numerous causes of immunosuppression that are not regarded in literature to date. One example is the use of immunosuppressive drugs or corticosteroids in autoimmune diseases or the use of such drugs for leprosy reactions. In the case of leprosy, the risk is not given by the disease itself but by the long-term immunosuppressive treatment that is sometimes necessary to control the leprosy reactions, which may put patients at risk of developing PJP.

## Main text

High-dose and long-term steroid therapies (such as 20mg prednisone or an equivalent dose for more than 4 weeks [4]) increase the risk of developing PJP in approximately 25% [5]. In leprosy, although corticosteroids are commonly used, the use of prophylaxis against PJP has not been contemplated in guidelines [6]. In this regard, it is important to highlight the importance of updated criteria for establishing prophylaxis with PJP due to the proliferation of immunosuppressive treatments and their widespread use in recent decades [4].

Up to 30-50% of patients with *M. leprae* infection have leprosy reactions during the course of their disease and high-dose steroids are usually required [7]. There are two types of leprosy reactions (LRs); cell-mediated type 1 LRs typically appear during the first months of therapy, especially in patients with borderline forms and they can occur all across the leprosy spectrum although are very rare in lepromatous leprosy.

They consist mainly of oedema, erythema, neuritis and ulceration of pre-existing skin lesions. Type 2 LRs, for which a mechanism secondary to systemic inflammation due to immunocomplex deposition is postulated, usually take place in patients with lepromatous and borderline-lepromatous leprosy in the form of skin eruptions or erythematous nodules that may ulcerate. Treatment mainly consists on high-dose steroids for at least 3-6 months, but it is usually necessary to extend it beyond 6 months or even to add other immunosuppressants such as methotrexate [7] or thalidomide. The latter is very effective in type 2 RL but restricted due to its teratogenicity, risk of neuropathy and CNS adverse effects. Therefore, these patients could be potential candidates for PJP prophylaxis. However, we have not found any cases of PJP in people with leprosy and prolonged steroid therapy in the literature, nor has this issue been addressed in leprosy guidelines to our knowledge.

Regarding the drug of choice for PJP prophylaxis, there is consensus that cotrimoxazole (SMX) should be the first line due to its efficacy and safety profile. However, in patients with intolerance or interactions, alternative drugs such as aerosolized pentamidine, atovaquone and dapsone have been proposed. Pentamidine was relegated from the first line of treatment as it was inferior to SMX and dapsone in patients with CD4+ lymphocytes <100/μL [8] and less effective in upper lobes due to ventilatory mechanics [2]. A safe and well-tolerated alternative could be atovaquone, although PJP mutations in cytochrome-b have been associated with therapeutic failure and it is an expensive drug. Dapsone, however, has generally shown similar efficacy to cotrimoxazole [2]. Although most studies were conducted mostly in the 1990s and HIV population, it has also shown similar efficacy to cotrimoxazole in a recent clinical trial in haematopoietic transplant patients [9].

The absence of reported cases of leprosy-associated PJP in patients with prolonged steroid treatment [7] may be due to the simultaneous use of dapsone as a treatment for this mycobacteriosis, a drug that would also act as a PJP prophylactic treatment. In fact, out of 33 patients with prolonged corticosteroid or methotrexate treatment included in different studies, two (6.1%) were not receiving dapsone [7, 10]. In this sense, about 4-8% of leprosy patients cannot be treated with dapsone, either because of toxicity, intolerance or antimicrobial resistance [7]. In addition, in some patients, RL persists beyond the end of leprosy treatment. Therefore, when high-dose or prolonged use of steroids or other immunosuppressants is necessary, the introduction of a drug active against *Pneumocystis jirovecii* as prophylaxis should be considered. If there have been no hypersensitivity reactions to dapsone, cotrimoxazole could be considered as it does not appear to increase the risk of haemolysis in the presence of glucose-6-phosphate dehydrogenase (G6PD) [11] deficiency compared to other alternatives that may be difficult to access or make available for economic reasons.

## Conclusions

Current indications for PJP prophylaxis outside oncological pathology and HIV infection are not well established, despite evidence of an increased risk of this complication in certain diseases associated with immunosuppression. Therefore, it should be considered to extend them and include patients under prolonged immunosuppressive treatment. In leprosy, there does not seem to be an increased incidence in PJP despite the need for long-term immunosuppressive therapies in certain patients. Among other reasons, the concomitant use of dapsone as a treatment for their underlying disease could act as a PJP preventive treatment. Thus, in patients who do not receive dapsone due to intolerance or antimicrobial resistance or in those who have already completed leprosy treatment but are still in need of immunosuppressive drugs, PJP prophylaxis (ideally with cotrimoxazole) should be considered. Otherwise, there should be a high index of suspicion for this complication in the presence of respiratory symptoms. Clinical studies in at-risk populations, other than PLWH, oncology and hematology patients, would be very useful to better understand PJP and improve preventive indications.

## Data Availability

Not applicable.

## Abbreviations

PJP: *Pneumocystis jirovecii* pneumonia
PLWH: Persons living with HIV
LRs: Leprosy reactions
SMX: Cotrimoxazole
G6PD: Glucosa-6-fosfato deshidrogenasa
